# Gα proteins Gvm2 and Gvm3 regulate vegetative growth, asexual development, and pathogenicityon apple in *Valsa mali*

**DOI:** 10.1371/journal.pone.0173141

**Published:** 2017-03-07

**Authors:** Na Song, Qingqing Dai, Baitao Zhu, Yuxing Wu, Ming Xu, Ralf Thomas Voegele, Xiaoning Gao, Zhensheng Kang, Lili Huang

**Affiliations:** 1 State Key Laboratory of Crop Stress Biology for Arid Areas and College of Plant Protection, Northwest A&F University, Yangling, People’s Republic of China; 2 Fachgebiet Phytopathologie, Institut für Phytomedizin, Universitӓt Hohenheim, Stuttgart, Germany; Austrian Institute of Technology, AUSTRIA

## Abstract

In fungi, heterotrimeric guanine-nucleotide binding proteins (G-proteins) are key elements of signal transduction pathways, which control growth, asexual and sexual development, as well as virulence. In this study, we have identified two genes encoding heterotrimeric G protein alpha subunits, named *Gvm2* and *Gvm3*, from *Valsa mali*, the causal agent of apple *Valsa* canker. Characterization of *Gvm2* and *Gvm3* mutants indicates that Gvm3 may be a crucial regulator of vegetative growth. Deletion of the corresponding gene results in a 20% reduction in growth rate. Besides, Gvm2 and Gvm3 seem to be involved in asexual reproduction, and mutants are hypersensitive to oxidative and cell membrane stresses. Interestingly, both G protein alpha subunits were most probably involved in *V*. *mali* virulence. In infection assays using *Malus domestica* cv. ‘Fuji’ leaves and twigs, the size of lesions caused by deletion mutants △*Gvm2*, or △*Gvm3* are significantly reduced. Furthermore, many genes encoding hydrolytic enzymes—important virulence factors in *V*. *mali*—are expressed at a lower level in these deletion mutants. Our results suggest that Gvm2 and Gvm3 play an important role in virulence probably by regulation of expression of cell wall degrading enzymes. △*Gvm2*, and △*Gvm3* mutants were further analyzed with respect to their impact on the transcript levels of genes in the cAMP/PKA pathway. The expression of the genes encoding adenylate cyclase *Vm*AC, protein kinase A (PKA) regulatory subunit *Vm*PKR, and PKA catalytic subunit *Vm*PKA1 are down-regulated in both mutants. Further analyses indicated that intracellular cAMP level and PKA activity are down-regulated in the △*Gvm3* mutant, but are basically unchanged in the △*Gvm2* mutant. Overall, our findings indicate that both Gvm2 and Gvm3 play diverse roles in the modulation of vegetative growth, asexual development, and virulence in *V*. *mali*.

## Introduction

Signal transduction cascades are the primary means by which external stimuli are communicated to the nuclei of eukaryotic organisms. In fungi, heterotrimeric guanine-nucleotide binding proteins (G-proteins) are key elements of signal transduction pathways, which control growth, asexual and sexual development, and virulence [1]. G-proteins are composed of three subunits: α, β and γ, which remain inactive in the heterotrimeric state with GDP bound to the Gα subunit [2]. Heterotrimeric G-proteins are activated by members of the seven-transmembrane-spanning family of receptors [3]. Binding of signal ligands to such receptors promotes an exchange of GDP to GTP on the Gα subunit, which then triggers a conformational change and dissociation from the Gβγ heterodimer [4]. Either Gα, or Gβγ, or both, are then free to activate downstream targets such as phospholipases, protein kinases, adenylate cyclases, or ion channels [5–8]. Activated G-proteins are later desensitized by the intrinsic GTPase activity of the Gα subunit, followed by re-association with the Gβγ complex. Therefore, the guanine nucleotide state of the Gα subunit seems to play a crucial role in controlling G-protein signaling [4].

Multiple alignments of fungal Gα genes revealed three major groups (I-III), based on amino acid sequence identity and functional similarities [9]. *Magnaporthe oryzae* for example possesses three Gα subunits (MagA, MagB, and MagC) with sequence similarity to mammalian Gα_s_, Gα_i_ superfamily proteins, and the fungal-specific GαII subfamily, respectively [10]. Deletion of *mag*A has no effect on vegetative growth, conidiation, or appressorium formation. Deletion of *mag*C reduces conidiation, but does not affect vegetative growth, or appressorium formation. However, disruption of *mag*B significantly reduces vegetative growth, conidiation, and appressorium formation [10]. In *Saccharomyces cerevisiae* Gpa2 regulates growth and pseudohyphal development via a cAMP-dependent mechanism [11,12]. Previous research showed that GanB plays a role during asexual conidiation and germination through regulating the cAMP/PKA pathway in response to glucose in *Aspergillus nidulans* [13,14]. Further fungal Gα subunit homologs that are well characterized include CPG-2 of *Cryphonectria parasitica* [15], and GNA-2 of *Neurospora crassa* [16], as well as FfG2 and FfG3 of *Fusarium fujikuroi* [17].

*Valsa* canker caused by the Ascomycete *Valsa mali*, is one of the most destructive diseases on apple in Eastern Asia [18], especially in China [19,20], Japan [21] and Korea [22], and leads to heavy damage to apple production [23,24]. Since the pathogen penetrates extensively into the host phloem and xylem [25], chemical treatment cannot effectively cure or control *Valsa* canker [23]. Once inside the host tissue the fungus can induce tissue maceration and cell death. Cell wall degrading enzymes have been shown to play an important role in the infection process of *V*. *mali* [25–28]. Meanwhile, it has been reported that G-protein signaling is implicated in the regulation of cellulase genes in *C*. *parasitica* and *Trichoderma reesei* [29–31]. Other studies have shown that Gα subunit homologs are important for regulating asexual development and virulence in *Botrytis cinerea* [32]. Therefore, a better understanding of the role of Gα subunits in pathogenesis and their regulatory pathways in *V*. *mali* seems crucial for developing more effective disease management strategies.

The genome sequence of *V*. *mali* opened new opportunities and perspectives for basic research to study its mechanism of plant infection [27]. To explore the roles of Gα subunits in *V*. *mali*, three Gα genes were cloned from the fungus, two of which were functionally characterized in the present study. We used Gα subunit mutants to elucidate their role in controlling development and virulence. We found that Gvm2 has a role in regulating conidiation and virulence. Gvm3 on the other hand seems to be involved in the regulation of vegetative growth, asexual development, and virulence. In addition, *Gvm2* and *Gvm3* also play a positive role in regulating cell wall degrading enzymes.

## Materials and methods

### Fungal strains, culture conditions

The wild type *V*. *mali* strain 03–8 [27] was obtained from the Laboratory of Integrated Management of Plant Diseases in College of Plant Protection, Northwest A&F University, PRC. All strains were routinely preserved in 20% glycerol at -80°C. Potato Dextrose Broth (PDB) was used to grow mycelium for DNA and RNA extraction. Fungal genomic DNA was extracted using the CTAB method [33]. The binary vector pBIG2RHPH2-GFP-GUS [34] was provided by Dr. Fengming Song at Zhejiang University, PRC. This vector carries the hygromycin B resistance cassette.

### Gene deletion and complementation analysis

To examine the biological function(s) of Gα proteins, we generated deletion mutants. To construct gene knockout cassettes the double joint PCR approach was used [35]. The hygromycin-phosphotransferase (*hph*) cassette, was amplified from PBIG2RHPH2-GFP-GUS with primers HYG/F and HYG/R (S1 Table). Upstream and downstream gene flanking sequences were amplified with primer pairs Gvm1-1F/Gvm1-2R, Gvm1-3F/Gvm1-4R, Gvm2-1F/Gvm2-2R, Gvm2-3F/Gvm2-4R, and Gvm3-1F/Gvm3-2R, Gvm3-3F/Gvm3-4R (S1 Table), respectively. After ligation with the *hph* cassette, the ligation product was transformed into *V*. *mali* strain 03–8 [36]. Hygromycin B (Roche, Mannheim, Germany) was added to a final concentration of 100 μg/ml for selection. Putative knockout mutants were identified by screening with primers GvmX-5F/GvmX-6R and H850/H852 (S1 Table), and further analyzed by PCR with primers GvmX-7F and H855R, and primers H856F and GvmX-8R (S1 Table). Southern Blot analyses were used to confirm gene replacement events (S2 Fig). Genomic DNA was labeled with digoxigenin (DIG)-dUTP using the DIG DNA Labeling and Detection Kit II (Roche). Hybridization and detection were carried out according to manufacturer’s instructions. All mutants generated in this study were preserved in 20% glycerol at -80°C.

For generation of complemented strains, fragments containing the entire Gα genes and their native promoter regions were amplified by PCR using primers Gvm2-GFP-CF/CR and Gvm3-GFP-CF/CR, respectively (S1 Table). The resulting PCR products were co-transformed into *S*. *cerevisiae* strain XK1-25 together with *Xho*I-digested pFL2 vector [37,38]. PGvm2-GFP and PGvm3-GFP fusion constructs were identified by PCR with primers Gvm2-GFP-CF/CR and Gvm3-GFP-CF/CR (S1 Table) and confirmed by sequencing. Fusion constructs were transformed into protoplasts of *V*. *mali* Δ*Gvm2* and Δ*Gvm3* mutants, respectively.

### Vegetative growth and pycnidia production

Small agar blocks were cut from the edge of 3-day-old cultures and placed onto Potato Dextrose Agar (PDA) in the dark at 25°C. Size and morphology of colonies were examined after 24 h, 48 h, and photographed after 48 h. For pycnidia production assays, cultures were grown in the dark for seven days, then transferred to an incubator with 12 h illumination per day at 25°C, and examined and photographed after 40 days. All treatments were performed with at least eight replicates, and all experiments were repeated three times. Data were analyzed by Student’s t-test using the SAS software package (SAS Institute, Cary, USA), p<0.05.

### Measuring stress responses

Fungal strains from glycerol stocks were inoculated onto PDA and placed in the dark for 3 d at 25°C. In order to test for sensitivity to osmotic stress, free radical stress, and cell membrane integrity, culture blocks of wild-type and the two mutants were inoculated onto PDA containing 0.1 M NaCl, 0.03% H_2_O_2_, or 0.01% SDS, respectively. Size and morphology of colonies were examined each day for three consecutive days. All treatments were performed with at least eight replicates, and the experiment was repeated three times. Data were analyzed by Student’s t-test using the SAS software package (SAS Institute), p<0.05.

### Quantitative RT-PCR

Infected twigs (0.2 g) were collected 6 h, 12 h, 18 h, 24 h, 36 h, and 48 h post inoculation (hpi), frozen in liquid nitrogen, and stored at -80°C. Mycelium was collected after 4 days incubation in PDB. Total RNA was extracted using the RNeasy Micro kit (Qiagen, Shenzhen, PRC). cDNA synthesis was performed using the StrataScript qPCR cDNA synthesis kit (Stratagene, La Jolla, U.S.A.) following the manufacturer’s instructions. Primers G6PDHF and G6PDHR [39] were used to amplify the 6-phosphogluconate dehydrogenase, decarboxylating (*G6PDH*) gene of *V*. *mali*. Relative changes in transcript level of target genes were calculated by the 2^–ΔΔCt^ method [40] with *G6PDH* as endogenous reference. Data from three biological replicates were used to calculate the mean and standard deviation. Primers used for qRT-PCR were listed in S2 Table.

### Pathogenicity tests

For leaf pathogenicity assays, leaves of *Malus domestica* Borkh. cv. ‘Fuji’ from the green house were collected and inoculated according to Wei et al. [41]. Leaves were washed with tap water, immersed in 0.6% sodium hypochlorite for 3 min and rinsed with sterile water three times. The basal parts of petioles were wrapped with moistened cotton. Four little wounds were made on a leaf using a sterile needle. A 5-mm PDA culture block was placed upside down onto each wound. Leaves were placed in a plastic box and the box was immediately covered with a vinyl film to retain humidity and placed in the dark at 25°C. Examination and photo documentation took place after 72 h. Assays were repeated eight times with three biological replicates each.

For twig pathogenicity assays, biennial intact apple twigs of *Malus domestica* Borkh. cv. ‘Fuji’ from the green house were collected and inoculated according to Wei et al. [41]. Twigs were cut into 40 cm long segments and washed with tap water, immersed in 1% sodium hypochlorite for 10 min and rinsed with sterile water three times. The top of the twigs was sealed with wax. Four wounds were made on each segment using a flat iron (5 mm diameter), wounds were 8 cm apart. Twigs were inserted into sand in a plastic basin. A 5-mm PDA culture block was used to inoculate each wound. Then the basin was immediately covered with a vinyl film to retain humidity and placed in the dark at 25°C. Examination and photo documentation took place after eleven days. All treatments were performed with at least eight replicates, and all experiments were repeated three times. Data were analyzed by Student’s t-test using the SAS software package (SAS Institute), p<0.05.

### cAMP and PKA activity assays

Three-day-old YEPD liquid mycelial cultures were harvested and frozen in liquid nitrogen. cAMP levels of the samples were measured using an HPLC [42,43]. Data were analyzed by Student’s t-test using the SAS software package (SAS Institute), p<0.05.

PKA activity was measured from 3-day-old YEPD liquid cultures. Sample (0.3g) were ground in liquid nitrogen. PKA activity was detected using PepTag^®^ Non-Radioactive Protein Kinase Assays kit (Promega, Madison, USA). Samples were separated on a 1.2% agarose gel at 160 V for 15 minutes.

## Results

### Identification of Gα subunit genes in *V*. *mali*

**Fig 1:**
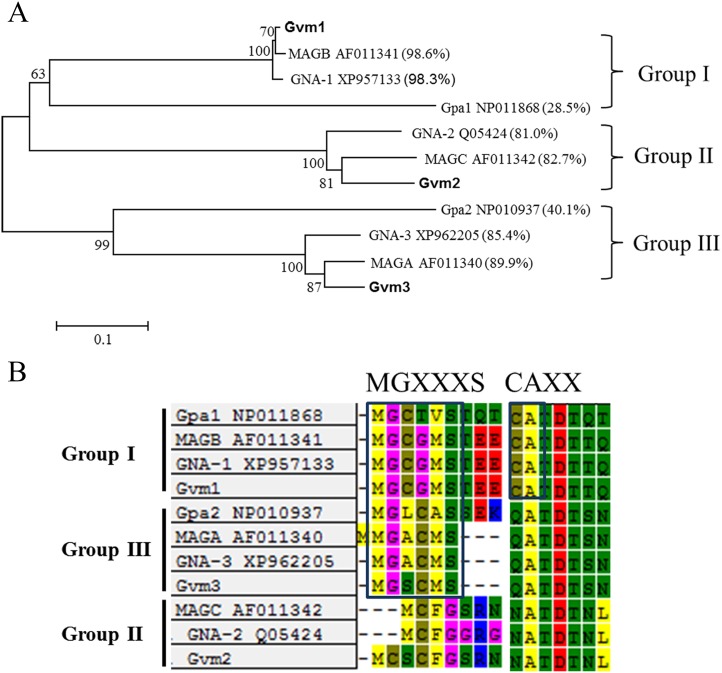
Three Gα subunits in *Valsa mali*. A: Phylogenetic analysis with G protein sequences from *V*. *mali*, *N*. *crassa*, *S*. *cerevisiae*, and *M*. *oryzae*. Protein sequences were aligned, and the Neighbor-Joining phylogenic tree was drawn using MEGA 5.0. B: Sequence alignment of the predicted active sites of the Gα subunits from *V*. *mali*, *N*. *crassa*, *S*. *cerevisiae*, and *M*. *oryzae*. MGXXXS: myristoylation site; CXXX: pertussis toxin-labeling site.

Based on sequence information from the *V*. *mali* genome, we cloned three Gα subunit genes termed *Gvm1* (VM1G_00876), *Gvm2* (VM1G_09956), and *Gvm3* (VM1G_04248) [27]. The *Gvm1* gene contains three introns and encodes a polypeptide of 353 amino acids, which shows 98.6% identity to *M*. *oryzae* MAGB (AF011341), and 98.3% to *N*. *crassa* GNA-1 (XP957133). *Gvm2* contains four introns and encodes a polypeptide of 357 amino acids, which shows 82.7% identity to *M*. *oryzae* MAGC (AF011342), and 81.0% to *N*. *crassa* GNA-2 (Q05424). *Gvm3* contains five introns and encodes a polypeptide of 355 amino acids, which shows 89.9% identity to *M*. *oryzae* MAGA (AF011340), 85.4% identity to *N*. *crassa* GNA-3 (XP962205), and 40.1% identity to *S*. *cerevisiae* Gpa2 (NP010937) (Fig 1A).

A phylogenetic tree of different Gα subunits shows three distinct groups: Group I, Group II, and Group III (Fig 1A), which correspond to the three groups proposed by Bӧlker [9]. Gvm1 contains a consensus myristoylation site (MGXXXS) [44], and pertussis toxin-labeling site (CXXX) [45] at its N and C termini, respectively. Gvm3 groups together with homologs in Group III. Gvm3 shows high similarity to mammalian Gα_s_ and contains a potential myristoylation site at its N terminus, but does not have a pertussis toxin-labeling site at its C terminus (Fig 1B). Gvm2 belongs to the fungal Gα subunit Group II and does not contain either site (Fig 1B).

### Deletion of *Gvm2* and *Gvm3*

Our results show that we successfully obtained knockout mutants for *Gvm2* and *Gvm3* (S1 Fig). All putative knockout mutants were also verified by Southern blot (S2 Fig). We obtained at least two deletion mutants for each gene with similar phenotypes, as described later in Table 1. For *Gvm1*, we failed to identify true knockout mutants after screening over one thousand transformants from at least four independent transformation experiments, indicating that deletion of this gene may be lethal.

**Table 1:** Wild type and mutant strains of *V*. *mali* used in this study.

Strains	Brief description	Reference
03–8	Wild-type	[27]
G2M-1	*gvm2* deletion mutant of 03–8	This study
G2M-2	*gvm2* deletion mutant of 03–8	This study
G2C-1	*gvm2*/*Gvm2* complemented transformant	This study
G2C-2	*gvm2*/*Gvm2* complemented transformant	This study
G3M-1	*gvm3* deletion mutant of 03–8	This study
G3M-2	*gvm3* deletion mutant of 03–8	This study
G3C-1	*gvm3*/*Gvm3* complemented transformant	This study
G3C-2	*gvm3*/*Gvm3* complemented transformant	This study

### *Gvm3* is involved in vegetative growth and asexual reproduction, whereas *Gvm2* only plays a role in asexual reproduction

**Fig 2:**
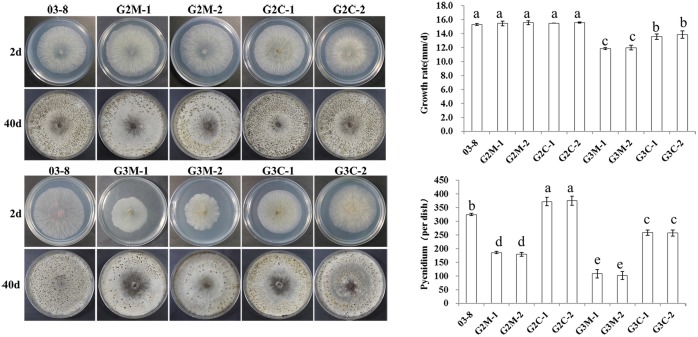
Colony morphology and conidiation of △*Gvm2* and △*Gvm3* deletion mutants. Colony morphology was assessed by incubating cultures in the dark for 48 h at 25°C, followed by measuring colony diameters. For the pycnidia production assays, cultures were placed in the dark at 25°C for 7 d, then transferred to the light, examined and photographed at 40 days. Bars indicate standard deviation of the mean of eight individual plates. All experiments were performed in triplicate. Different letters indicate statistical significance.

Gvm3 was found to play an important role in vegetative growth. Deletion of this gene results in an over 20% reduction in growth rate (Fig 2). Compared with the wild type strain (15.3 mm/day), growth rates of △*Gvm3* mutants G3M-1 (11.9 mm/day) and G3M-2 (12.0 mm/day) are significantly reduced (p = 0.05). Both complemented strains (G3C-1 and G3C-2) exhibit at least partially restored growth rates. However, both △*Gvm2* mutants (G2M-1 and G2M-2) show unaltered growth (Fig 2). In addition, Gvm2 and Gvm3 seem to be involved in asexual reproduction. Compared with the wild type strain, the amount of conidiation of △*Gvm2* and △*Gvm3* mutants is significantly decreased. Complementation (G2C-1, G2C-2 and G3C-1, G3C-2) could at least partially restore normal conidiation.

### Susceptibility of △*Gvm2* and △*Gvm3* mutants to abiotic stresses

**Fig 3:**
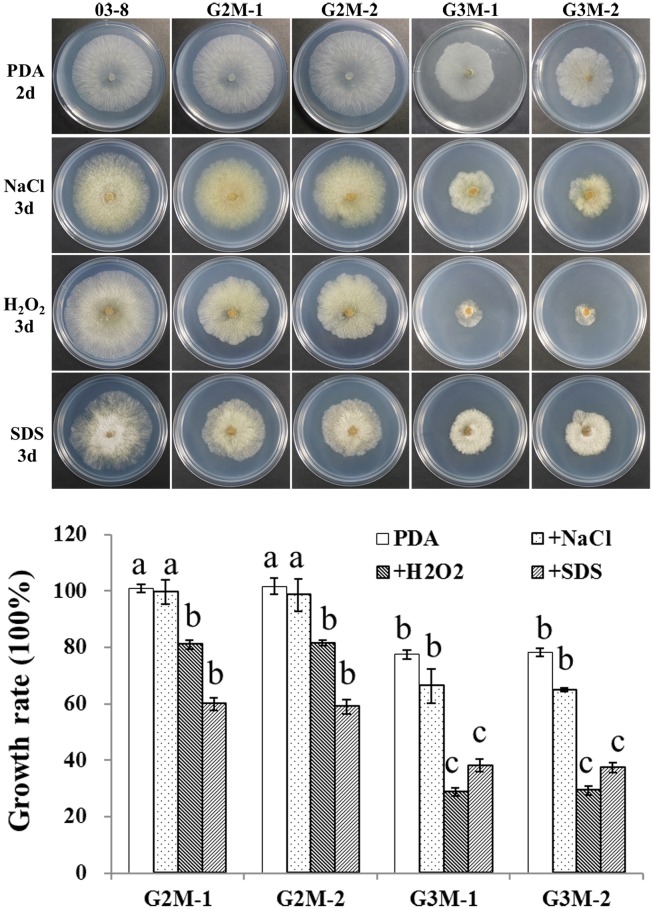
Responses of △*Gvm2* and △*Gvm3* mutants to hyperosmotic, oxidative, and membrane stresses. Colony diameters of wild-type strain 03–8, △*Gvm2* mutants G2M-1, G2M-2 and △*Gvm3* mutants G3M-1, G3M-2 on PDA with 0.1 M NaCl, 0.03% H_2_O_2_, or 0.01% SDS were measured after incubation in the dark for 3 d at 25°C. The percentage of the growth rate of the G2M-1, G2M-2, G3M-1, and G3M-2 mutants compared to that of the wild-type (set at 100%) on PDA cultures with or without different stresses. Different letters indicate statistically significant differences (P < 0.05, T-test). Bars indicate standard deviation of the mean of eight individual plates. All experiments were performed in triplicate.

As results from growth on PDA supplemented with 0.03% H_2_O_2_ suggest, △*Gvm2* and △*Gvm3* mutants seem to be more sensitive to Reactive Oxygen Species (ROS) (Fig 3). When assayed for growth on PDA plates supplemented with 0.01% SDS, to simulate membrane stress, both mutants, especially △*Gvm3* mutants, showed drastically reduced growth. By contrast, △*Gvm2* and △*Gvm3* mutants do not seem to be affected by osmotic stress, simulated by inclusion of 0.1 M NaCl to PDA (Fig 3). These results indicate that Gvm2 and Gvm3 may play the same role with respect to tolerance to abiotic stresses in *V*. *mali*.

### Deletion of *Gvm2*, or *Gvm3* leads to reduced virulence

**Fig 4:**
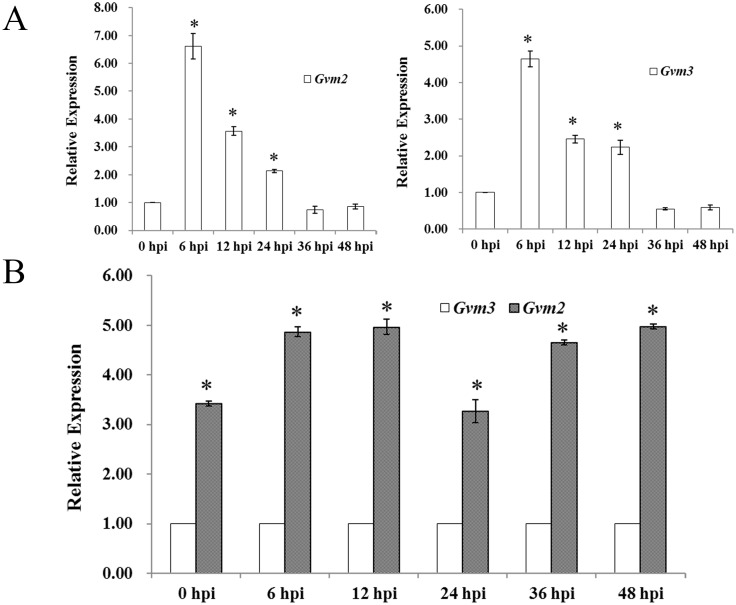
Transcript levels of *Gvm2* and *Gvm3* assayed by qRT-PCR. A: RNA samples were isolated from mycelium of strain 03–8 cultured in PDB medium at 25°C for 48 h. 0 hpi: axenic culture. Infected twigs were collected 6, 12, 24, 36 and 48 hpi. Relative transcript levels of *Gvm2* and *Gvm3* were calculated with *G6PDH* as internal control using the 2^–ΔΔCT^ method. Transcript levels of *Gvm2* or *Gvm3* at the mycelium stage were set to 1. B: Relative transcript levels of *Gvm2* in comparison with *Gvm3*. The transcript level of *Gvm3* was set to 1 for all samples. Data from three biological replicates were used to calculate the mean and standard deviation.

To gain insight into a possible function of Gvm2 and Gvm3 in pathogenicity, we examined their transcription profiles during infection using quantitative real-time PCR (qRT-PCR). Compared to axenically grown mycelium, transcript levels of *Gvm2* are 6.6-fold, 3.6-fold, and 2.1-fold higher at 6 hpi, 12 hpi, and 24 hpi, respectively. However, expression of *Gvm2* is not significantly changed at 36 hpi and 48 hpi (Fig 4A). Similarly, transcript levels of *Gvm3* are 4.7-fold, 2.5-fold, and 2.2-fold increased at 6 hpi, 12 hpi, 24 hpi, respectively (Fig 4A). These results confirm that transcripts of *Gvm2* and *Gvm3* are up-regulated during early stages of infection. *Gvm3* has a transcript profile similar to *Gvm2*. However, its transcript levels in general are lower than those of *Gvm2* (Fig 4B).

**Fig 5:**
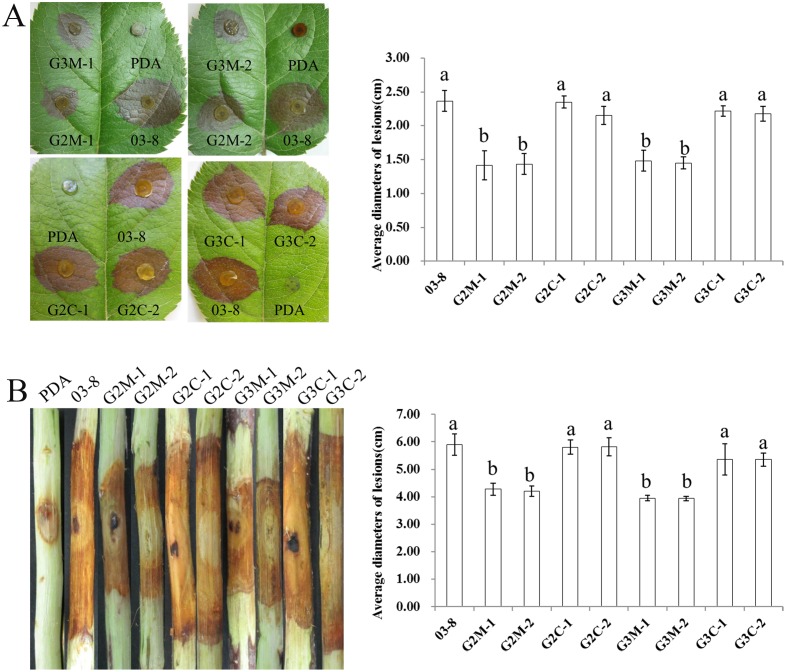
Assay for plant infection of the △*Gvm2* and △*Gvm3* mutants. 5 mm circular agar plugs were inoculated onto (A) leaves or (B) twigs, examined and photographed 3, and 11 dpi, respectively. Three biological replicates and eight technical replicates were performed. Bars indicate standard deviation of the mean of eight individual plants.

To further characterize the function of Gvm2 and Gvm3 in pathogenesis, △*Gvm2* and △*Gvm3* mutants were inoculated onto leaves and twigs. In infection assays with apple leaves, virulence of both deletion mutants is significantly reduced. Compared to the wild type strain 03–8, the average diameter of lesions caused by △*Gvm2*, or △*Gvm3* mutants are significantly decreased. Compared to the wild type (23.7 mm), △*Gvm2* mutants show a reduction by 40.1%, and △*Gvm3* mutants exhibit a reduction of 35.0% (Fig 5A). Similarly, lesion lengths caused by △*Gvm2* and △*Gvm3* mutants are also smaller on twigs. The lesion length of wild type 03–8 strain was 59.0 mm. Compared to the wild type, both of △*Gvm2* mutants G2C-1 and G2C-2 show a reduction of 27.5% and 28.8%, and △*Gvm3* mutants G3C-1 and G3C-2 exhibit a reduction of 33.1% and 33.2% (Fig 5B).

**Fig 6:**
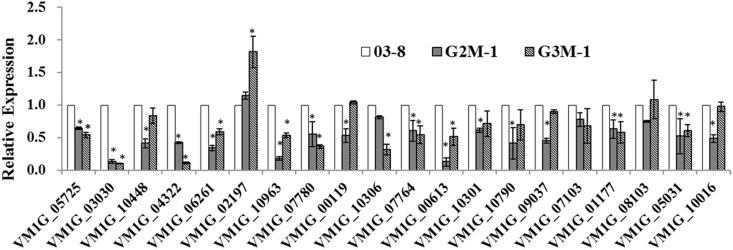
Transcript levels of Cell Wall Degrading Enzyme (CWDE) genes in the △*Gvm2* and △*Gvm3* mutants of *V*. *mali*. RNA samples were isolated from infected twigs of WT, G2M-1, and G3M-1 three days after inoculated at 25°C. Relative transcript levels were calculated with *G6PDH* as internal control using the 2^–ΔΔCT^ method. Transcript levels of WT were set to 1 for all samples.

Cell wall degrading enzymes have been shown to constitute important virulence factors in *V*. *mali* [25–28]. A role for G protein signaling in cellulase gene expression was described in *C*. *parasitica* and *T*. *reesei* [29–31]. To further analyze the influence of Gvm2, or Gvm3 on cell wall degrading enzymes, transcript levels of different genes, including nine pectinase, six cellulase and five hemicellulase genes (S3 Table) encoded in the *V*. *mali* genome were checked in △*Gvm2* and △*Gvm3* mutants. RNA samples were isolated from vegetative mycelium of wild type strain 03–8, △*Gvm2*, and △*Gvm3* mutants inoculated on twigs for 3 days at 25°C. Our results show that many hydrolytic enzyme encoding genes including pectinase, cellulase and hemicellulase genes are expressed at much lower levels in △*Gvm2* and △*Gvm3* mutants (Fig 6). These results clearly indicate that Gvm2 and Gvm3 are involved in regulating cell wall degrading enzyme genes.

### *Gvm2* and *Gvm3* mutants’ influence on interrelated downstream genes

**Fig 7:**
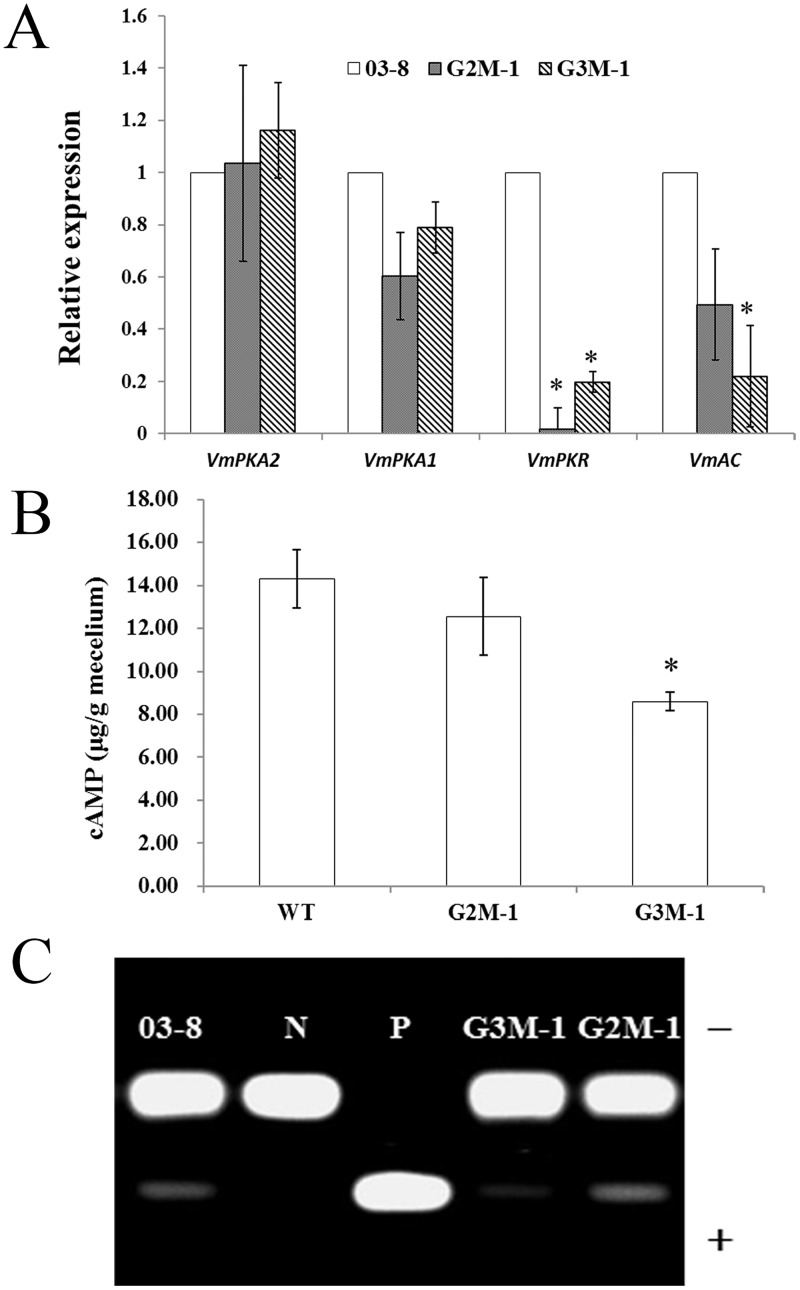
Assay for cAMP/PKA signaling pathway of △*Gvm2* and △*Gvm3* mutants. A: Transcript levels of genes related to the cAMP/PKA signaling pathway. Relative transcript levels of *VmPKA1*, *VmPKA2*, *VmPKR* and *VmAC* genes in the wild type strain were set to 1. B: Intracellular cAMP levels in the wild-type and mutants. Bar chart showing quantification of intracellular cAMP in the mycelia of the indicated strains following three days of culturing in Yeast Extract Peptone Dextrose Medium (YEPD). The standard deviation represent SD of three replicates. C: PKA activity in the wild-type and mutants. Phosphorylated (+), nonphosphorylated (-), cAMP-Dependent Protein Kinase, Catalytic Subunit was the PKA positive control (P); water insteaded of PKA added as the negative control (N).

To further analyze the effects of a deletion of *Gvm2* or *Gvm3* on the expression of genes of the cAMP/PKA pathway, we checked the transcript levels of different genes, including one adenylate cyclase gene (*VmAC*, VM1G_01407), two PKA catalytic subunits genes (*VmPKA1*, VM1G_00266; and *VmPKA2*, VM1G_08687), and one PKA regulatory subunit gene (*VmPKR*, VM1G_08329) encoded in the *V*. *mali* genome. RNA samples were isolated from vegetative mycelium of wild type strain 03–8, △*Gvm2* and △*Gvm3* mutants cultured in PDB medium for 4 d at 25°C. Our results show that *VmAC*, and *VmPKR* are down-regulated in both mutants (Fig 7A). The expression of *VmPKA1* is also down-regulated, however, *VmPKA2* is not significantly affected in the two mutants (Fig 7A).

### Effects of *V*. *mali* Gvm2 and Gvm3 on cAMP level and PKA activity

It was reported that Group III Gα proteins could influence cAMP levels through the regulation of adenylate cyclase [9]. To determine whether Gvm2 and Gvm3 are also involved in this process, we measured intracellular cAMP levels in the mutants G2M-1 and G3M-1. Our results indicate that the △*Gvm3* mutant strain G3M-1 accumulates somewhat lower levels of cAMP than the wild-type strain. Compared with the wild-type, G3M-1 shows a 1.7-fold lower intracellular cAMP level, while the intracellular cAMP level in G2M-1 did not obviously decrease (Fig 7B). These results suggest that Gvm3 protein plays a role in regulating the intracellular cAMP level.

Similarly, PKA activity is reduced in the △*Gvm3* mutant strain G3M-1 (Fig 7C). However, PKA activity in the △*Gvm2* mutant strain G2M-1 shows no obvious change (Fig 7C). These results suggest that Gvm3 is also involved in regulating PKA activity.

## Discussion

The importance of G protein signaling in regulating diverse biological processes in fungi has already been demonstrated [46]. In this study, we have identified genes encoding two heterotrimeric Gα subunits, *Gvm2* and *Gvm3*, from *V*. *mali*. We found that Gvm2 and Gvm3 play various roles in the modulation of vegetative growth, asexual development, and virulence possibly via the cAMP/PKA pathway in this pathogenic fungus. Reduced virulence of △*Gvm2* and △*Gvm3* mutants may be due to a lower expression level of cell wall degrading enzymes.

Except for yeasts, which contain two Gα proteins, most characterized filamentous fungi possess three Gα proteins belonging to distinct groups [9,47]. Group I Gα proteins are highly conserved in most filamentous fungi, containing a consensus sequence for myristoylation (MGXXXS) at the amino terminus [44,48] and a site for ADP-ribosylation by pertussis toxin (CAAX) at the carboxy terminus [45]. Gvm1 seems to be a Group I Gα subunit, but unfortunately, we were not able to obtain a deletion mutant. Gvm2 is a Group II Gα subunit, and Gvm3 is a Group III Gα subunit similar to *M*. *oryzae* MAGC, and MAGA, respectively. Group II Gα subunits are not as well conserved as Group I, or III Gα subunits. Group III Gα subunits are highly conserved and most possess a myristoylation sequence [49]. We found that Gvm3 contains a potential consensus myristoylation site (MMGXXXS) at its N terminus, while Gvm2 does not contain such a site.

Gvm3 was found to play an important role in the regulation of vegetative growth. Similarly, deletion of *ffg3* in *F*. *fujikuroi* caused reduces growth rates on minimal as well as complete medium [17]. Defects in growth rate upon deletion of Gα subunit genes has also been shown for *A*. *nidulans*, and *B*. *cinerea* [13,14,32]. However, deletion of *mag*A in *M*. *oryzae* has no effect on vegetative growth [10]. It remains to be clarified if there is a species-specific pattern in the influence of Group III Gα subunits on the growth of different fungi. Our △*Gvm2* mutants on the other hand exhibit no significant effect on fungal growth. It is reported that the role of Group II Gα subunits in vegetative growth is less explicit. For example, deletion of *mag*C does not affect vegetative growth in *M*. *oryzae* [10].

Gα subunits have also been reported to be involved in asexual reproduction. Deletion of *mag*A has no effect on conidiation, but a mutation in *mag*C considerably reduces conidiation in *M*. *oryzae* [10]. In this study, we found that conidiation of *V*. *mali* was negatively influenced by mutations in both Gα subunit genes, *Gvm2* and *Gvm3*. Despite the different growth rate phenotypes exhibited by these mutants, both Gα subunits seem to contribute to the regulation of asexual reproduction in *V*. *mali*.

We also found that mutants defective in *Gvm2*, or *Gvm3* are more sensitive to free radicals. Some reports have indicated that G protein-coupled signaling components are involved in H_2_O_2_-induced responses [50]. Besides, it has been reported that Gα_i_ and Gα_o_ are critical components of oxidative stress responses, e.g. for activation of extracellular signal-related kinase (ERK) [50]. Based on our findings, we suggest that Gvm2 as well as Gvm3 play important roles in oxidative stress responses.

It has also been shown that G proteins may play a significant role in pathogenesis [49]. The results from our qRT-PCR analyses show that transcript levels of *Gvm2* and *Gvm3* are up-regulated during early stages of infection. Both proteins may therefore play an important role in the early stages of infection in *V*. *mali*. Many reports indicate that Group III Gα subunits may be involved in pathogenesis. In *Ustilago maydis*, Gpa3 seems to be involved in the invasion of corn [51]. Group III Gα subunits in *B*. *cinerea* (Bcg3), *Cryptococcus neoformans* (Gpa1), *F*. *fujikuroi* (Ffg3) and *Fusarium oxysporum* (Fga2) also seem to be required for full virulence [17,32,52–55]. However, deletion of *mag*A in *M*. *grisea* does not appear to affect the ability to infect and spread within host tissue [10]. In infection assays with apple leaves and twigs, virulence of *Gvm3* deletion mutants is significantly reduced (Fig 5). We cannot totally exclude that the growth defect of △*Gvm3* mutants may contribute to the reduced virulence, at least partially. However, △*Gvm3* mutants show a 30% to 35% reduction in lesion length in leaves or twigs (Fig 5)—this is not proportional to the 20% reduction in growth rate (Fig 2). In addition, cell wall degrading enzymes have been described as important virulence factors in *V*. *mali* [25–28]. It is likely that the defect in virulence of △*Gvm3* mutants may be related to its reduced expression of cell wall degrading enzyme genes (Fig 6). G protein signaling in cellulase gene expression has also been described for *C*. *parasitica* and *T*. *reesei* [29–31]. Interestingly, △*Gvm2* mutants, which do not show a growth defect, also show a significant reduction in virulence. Reduced virulence of △*Gvm2* mutants is consistent with reduced expression of cell wall degrading enzymes, indicating that Gvm2 also plays an important role in virulence. Transcript levels of genes encoding cell wall degrading enzymes in △*Gvm2* mutants are lower compared to △*Gvm3* mutants. This may be the reason why △*Gvm2* mutants show the same reduction in virulence as △*Gvm3* mutants, though △*Gvm2* mutants do not exhibit a growth defect. Similarly, it has been found that Gpa3 from *C*. *neoformans* is also involved in pathogenesis [54]. However, in most organisms, the function of Group II Gα proteins is less significant than that of Group III Gα proteins. For example, deletion of *mag*C in *M*. *oryzae*, *ffg*3 in *F*. *fujikuroi*, and *gpa*2 in *U*. *maydis* has no effect on virulence [10,17,51] and *B*. *cinerea bcg-2* mutants only show slightly reduced virulence [32].

Both Gβγ and Gα subunits are able to trigger downstream signaling pathways by interacting with various targets such as phosphodiesterases, protein kinases, and adenylate cyclases [6,56,57]. In this study, we analyzed transcript levels of *VmAC*, *VmPKR*, *VmPKA1*, and *VmPKA2* in △*Gvm3* and △*Gvm2* mutants. Transcript levels of *VmAC*, *VmPKR*, and *VmPKA1* are down-regulated in both mutants. Gvm2 and Gvm3 may therefore be involved in the cAMP/PKA pathway. To further determine whether Gvm2 and Gvm3 proteins are involved in this process, we measured intracellular cAMP levels. Our results indicate that △*Gvm3* mutants accumulate somewhat lower levels of cAMP, while the intracellular cAMP level in △*Gvm2* mutants seems unchanged (Fig 7B). We also measured PKA activity in △*Gvm2* and △*Gvm3* mutants. Results show that PKA activity is reduced in △*Gvm3* mutants (Fig 7C). However, PKA activity in △*Gvm2* mutants show no change (Fig 7C). These results suggest that Gvm3 plays a more important role in regulating the cAMP-PKA signaling pathway. It has been reported that Group III Gα proteins could influence cAMP levels through the regulation of adenylate cyclase [9]. Gα proteins, including *A*. *nidulans* GanB, *U*. *maydis* Gpa3, *F*. *fujikuroi* Ffg3 and *C*. *neoformans* Gpa1, hae been implicated in the regulation of cAMP signaling [14,17,52,53,58,59].

In conclusion, two heterotrimeric Gα subunits, Gvm2 and Gvm3, were functionally characterized in *V*. *mali*. Both seem to be important for virulence. Gvm3 also seems to be involved in regulating vegetative growth. Both, Gvm2 and Gvm3, seem to be involved in the response to different abiotic stresses in *V*. *mali*.

## Supporting information

S1 Fig Gene knockout by homologous recombination and PCR screening of transformants with four primer pairs. Verification of mutants by PCR was done using four pairs of primers. Primers GvmX-5F and GvmX-6R (1) are located within the ORFs for negative screening, primers H852 and H850 (2) are located within the hygromycin-resistant gene, primers GvmX-7F and GvmX-8R are located beyond the gene flanking sequences, primers H856F and H855R are located within the hygromycin-resistance conferring gene, primers GvmX-7F/H855R (3) for positive screen (upstream), and primers H856F/ GvmX-8R (4) for positive screen (downstream). M: 2,000 bp marker.
(TIF)

S2 Fig Strategy of knocking out Gα subunit genes in V. mali. Arrows indicate orientations of the Gα and hygromycin phosphotransferase (hph) genes. Thin lines below the arrows indicate the probe sequence for each gene (Probe 1), or the hph gene (Probe 2). A: Southern blot analyses of Gvm2 knockout mutants. Genomic DNA was digested with restriction enzymes HindIII (H), EcoRI (EI), or XbaI (X). When hybridized with a Gvm2 fragment amplified with primers Gvm2-5F/Gvm2-6R (Probe 1), the wild-type strain 03–8 shows the expected 4.4 kb band. gvm2 mutants show no corresponding hybridization signal. When hybridized with an hph probe (Probe 2) amplified with primers H850/H852, the wild-type strain shows no hybridization signal. gvm2 mutants on the other hand exhibit the expected 4.6 kb, or 9.0 kb bands in the respective XbaI, or EcoRI digests. B: Southern blot analyses of Gvm3 knockout mutants. Genomic DNA was digested with EcoRV (EV), or BamHI (B). When hybridized with a Gvm3 fragment amplified with primers Gvm3-5F/Gvm3-6R (Probe 1), the wild-type strain 03–8 shows the expected 4.1 kb band. gvm3 mutants show no hybridization signal. When hybridized with an hph probe (Probe 2) amplified with primers H850/H852, the wild-type strain shows no hybridization signal, whereas the gvm3 mutants show the expected 4.0 kb band.
(TIF)

S1 Table Primers for gene knockout cassette establishment and detection. (DOCX)

S2 Table Primers for detection of relative expression levels. (DOCX)

S3 Table Primers used for melanin biosynthesis related genes and cell wall-degrading enzyme genes expression. (DOCX)
