# Supplementary figures and images for: Gα proteins Gvm2 and Gvm3 regulate vegetative growth, asexual development, and pathogenicityon apple in Valsa mali
Source: PLoS One. 2017 Mar 7;12(3):e0173141. doi: 10.1371/journal.pone.0173141 (PMC5340391; doi:10.1371/journal.pone.0173141)

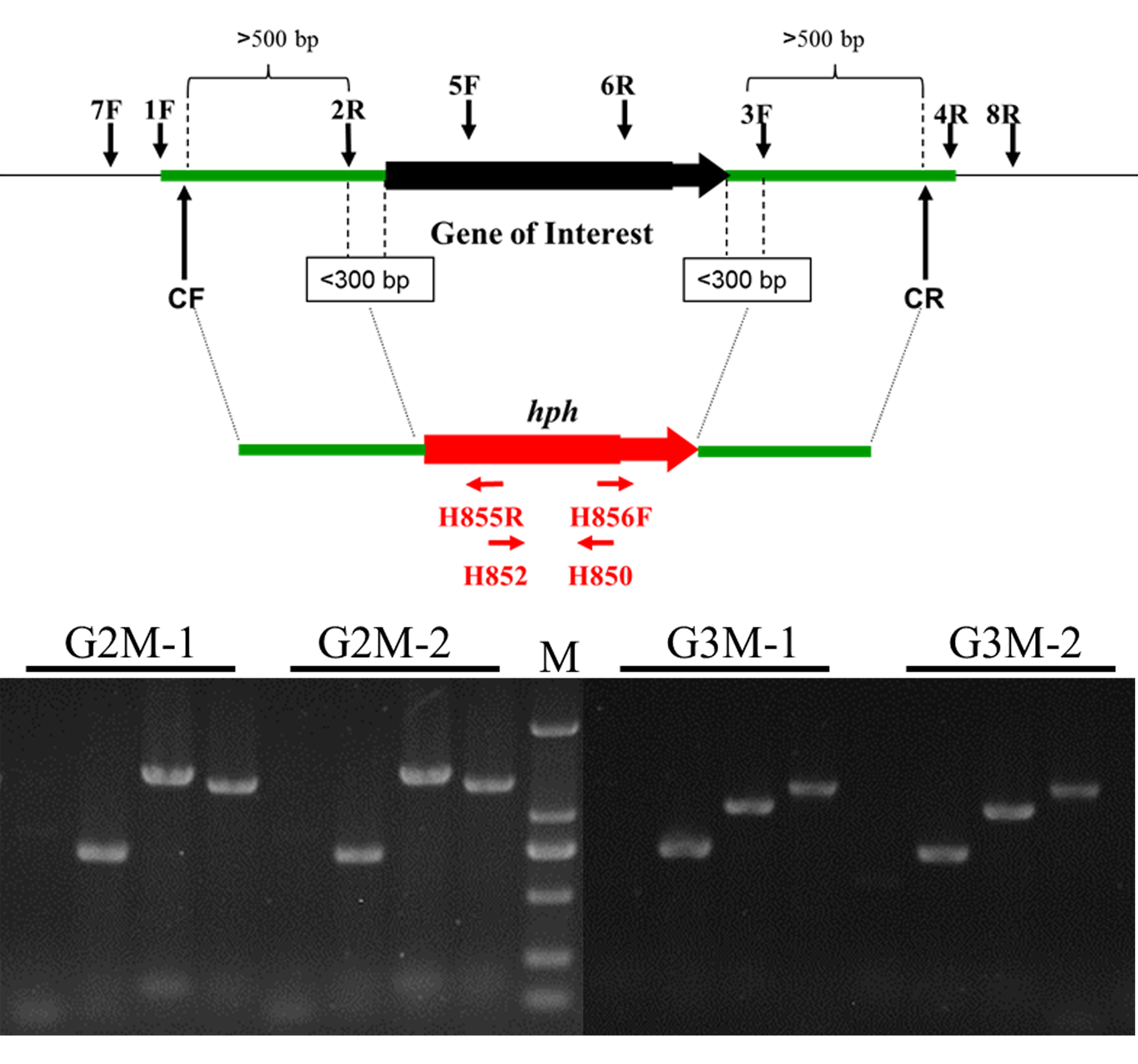

Supplement: S1 Fig — Verification of mutants by PCR was done using four pairs of primers. Primers GvmX-5F and GvmX-6R (1) are located within the ORFs for negative screening, primers H852 and H850 (2) are located within the hygromycin-resistant gene, primers GvmX-7F and GvmX-8R are located beyond the gene flanking sequences, primers H856F and H855R are located within the hygromycin-resistance conferring gene, primers GvmX-7F/H855R (3) for positive screen (upstream), and primers H856F/ GvmX-8R (4) for positive screen (downstream). M: 2,000 bp marker. (TIF) [file pone.0173141.s001.tif]

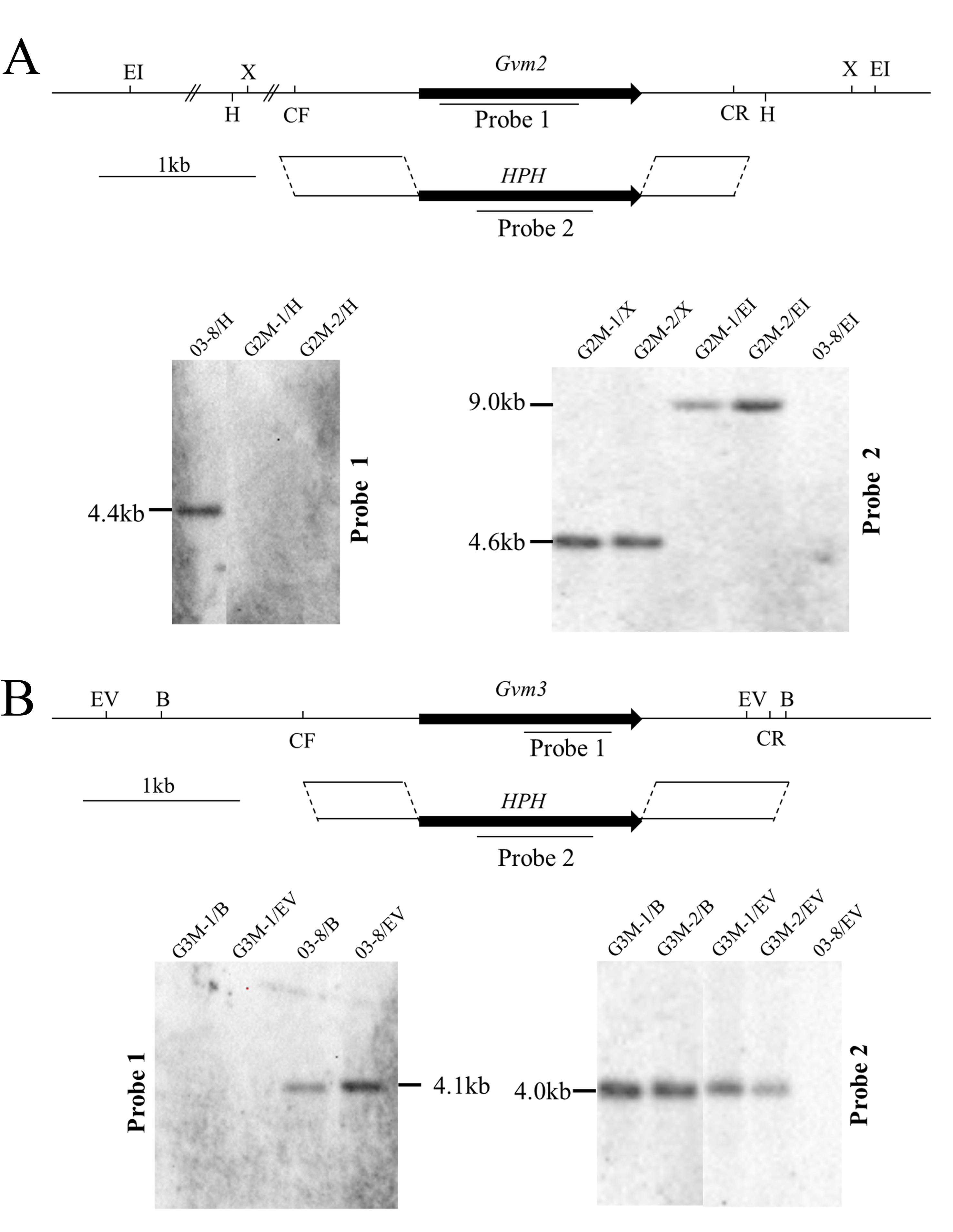

Supplement: S2 Fig — Arrows indicate orientations of the Gα and hygromycin phosphotransferase (hph) genes. Thin lines below the arrows indicate the probe sequence for each gene (Probe 1), or the hph gene (Probe 2). A: Southern blot analyses of Gvm2 knockout mutants. Genomic DNA was digested with restriction enzymes HindIII (H), EcoRI (EI), or XbaI (X). When hybridized with a Gvm2 fragment amplified with primers Gvm2-5F/Gvm2-6R (Probe 1), the wild-type strain 03–8 shows the expected 4.4 kb band. gvm2 mutants show no corresponding hybridization signal. When hybridized with an hph probe (Probe 2) amplified with primers H850/H852, the wild-type strain shows no hybridization signal. gvm2 mutants on the other hand exhibit the expected 4.6 kb, or 9.0 kb bands in the respective XbaI, or EcoRI digests. B: Southern blot analyses of Gvm3 knockout mutants. Genomic DNA was digested with EcoRV (EV), or BamHI (B). When hybridized with a Gvm3 fragment amplified with primers Gvm3-5F/Gvm3-6R (Probe 1), the wild-type strain 03–8 shows the expected 4.1 kb band. gvm3 mutants show no hybridization signal. When hybridized with an hph probe (Probe 2) amplified with primers H850/H852, the wild-type strain shows no hybridization signal, whereas the gvm3 mutants show the expected 4.0 kb band. (TIF) [file pone.0173141.s002.tif]
