# Supplementary material for: Gα proteins Gvm2 and Gvm3 regulate vegetative growth, asexual development, and pathogenicityon apple in Valsa mali
Source: PLoS One. 2017 Mar 7;12(3):e0173141. doi: 10.1371/journal.pone.0173141 (PMC5340391; doi:10.1371/journal.pone.0173141)
Supplement: S1 Table — (DOCX) [file pone.0173141.s003.docx]

**S1 Table. Primers for gene knockout cassette establishment and detection**

| **Primers** | **Sequences (5’-3’)** |
| --- | --- |
| Gvm1-1F | AAAGAGGATGGCTGAGGTTGA |
| Gvm1-2R | *TTGACCTCCACTAGCTCCAGCCAAGCC*AATGTTTGTTTATCGGTTCGC |
| Gvm1-3F | *GAATAGAGTAGATGCCGACCGCGGGTT*TCGCACGATTTCTTCCTTACG |
| Gvm1-4R | GTCAGGACAGCCACTACGAG |
| Gvm1-CF | CCTGTTACTGCTTGTTCCTGA |
| Gvm1-CR | ACGGTTTGGTTTGGGTTTACA |
| Gvm1-5F | TTTCAGCAACACCGTCCAAT |
| Gvm1-6R | GTAGTCTTCGCCACCTTCGT |
| Gvm1-7F | GGAGTGAAAGGTAACACGGCT |
| Gvm1-8R | CGGGGTTGATGAGGATGA |
| Gvm2-1F | CCGTGATCGCCGTTTGAGAA |
| Gvm2-2R | *TTGACCTCCACTAGCTCCAGCCAAGCC*CCCTTGAGGCGGGAGATGTG |
| Gvm2-3F | *GAATAGAGTAGATGCCGACCGCGGGTT*CCCACGCGAACCTGCTTTAC |
| Gvm2-4R | GAACCGAGCAATACGGAACG |
| Gvm2-CF | ACCCGCAATCCCGTACCATA |
| Gvm2-CR | GTCTTGTGGCTCCTCGGCTC |
| Gvm2-5F | GTCAACTCCATCCATCCGTCA |
| Gvm2-6R | CCTCGTTCATTTGGTTCTTCC |
| Gvm2-7F | AAGGCATCAGGACGTGAGTG |
| Gvm2-8R | ACCCGTACAGGTATGCTCAGGTTT |
| Gvm3-1F | TCATTCTGTCTCTCCTCTCG |
| Gvm3-2R | *TTGACCTCCACTAGCTCCAGCCAAGCC*TTGTCTGGTATCGGTGGCTC |
| Gvm3-3F | *GAATAGAGTAGATGCCGACCGCGGGTT*ATGTCTACGAGCGGTCTTT |
| Gvm3-4R | AATCAGACTGTCAGGAGGC |
| Gvm3-5F | TGCCCAACGAGATGGATGT |
| Gvm3-6R | GATTGTTTGCCACCGCCTA |
| Gvm3-CF | CTTGGCACAAAAAGACTGGG |
| Gvm-CR | TACACGCCCAGACGAGAAA |
| Gvm3-7F | CTGCCTTGTCCCCATCTC |
| Gvm3-8R | GCCTTTCGCACTTCCTTCA |
| HYG/F | GGCTTGGCTGGAGCTAGTGGAGGTCAA |
| HYG/R | AACCCGCGGTCGGCATCTACTCTATTC |
| H850 | ATGTTGGCGACCTCGTATTGG |
| H852 | TTCCTCCCTTTATTTCAGATTCAA |
| H855R | GCTGATCTGACCAGTTGC |
| H856F | GTCGATGCGACGCAATCGT |
| Gvm2-GFP-CF | *CGACTCACTATAGGGCGAATTGGGTACTCAAATTGG*CTACTTGCTATTTTGGGGTTTG |
| Gvm2-GFP-CR | *CACCACCCCGGTGAACAGCTCCTCGCCCTTGCTCAC*AAGAATCAGCTGCTTCAGATTTC |
| Gvm3-GFP-CF | *CGACTCACTATAGGGCGAATTGGGTACTCAAATTGG*GGACATTGAGCGGTTGAG |
| Gvm3-GFP-CR | *CACCACCCCGGTGAACAGCTCCTCGCCCTTGCTCAC*TAGAATACCAGAGTCTTTTAAAG |
