# Supplementary material for: Gα proteins Gvm2 and Gvm3 regulate vegetative growth, asexual development, and pathogenicityon apple in Valsa mali
Source: PLoS One. 2017 Mar 7;12(3):e0173141. doi: 10.1371/journal.pone.0173141 (PMC5340391; doi:10.1371/journal.pone.0173141)
Supplement: S2 Table — (DOCX) [file pone.0173141.s004.docx]

**S2 Table. Primers for detection of relative expression levels**

| **Sequence number** | **Primers**  **name** | **Sequences (5’-3’)** | **Annotation** | **Reference** |
| --- | --- | --- | --- | --- |
|  | G6PDH-F | TCAGAACAAGTTCGAGGGCGACAA | 6-phosphogluconate dehydrogenase,  decarboxylating | (Yin et al, 2013) |
|  | G6PDH-R | TGAGGGCAATAGAGGGCTTGTTCA |  |  |
| VM1G_09956 | Gvm2-Q-F | CAAAGAGCCCCATCACACAA | Guanine nucleotide-binding protein alpha-2 subunit | (Yin et al, 2015) |
|  | Gvm2-Q-R | AAGGTTCGTATCGGTAGCGTT |  |  |
| VM1G_04248 | Gvm3-Q-F | CGACTACCTGCCCAACGAG | Guanine nucleotide-binding protein alpha-3 subunit |  |
|  | Gvm3-Q-R | GAATCCACTTCTTCCGCTCAC |  |  |
| VM1G_01407 | VmAC-Q-F | GAACTACGCTTGCTGAACCTCT | Adenylate cyclase |  |
|  | VmAC-Q-F | TCCGCAGTCTAACACCGCTAA |  |  |
| VM1G_00266 | VmPKA1-Q-F | GCTACAACGCAATCGCTAA | cAMP-dependent protein kinase catalytic subunit |  |
|  | VmPKA1-Q-R | CGAGTATTTCCCTTTGGTCT |  |  |
| VM1G_08687 | VmPKA2-Q-F | ACCTCAAGCCCGAGAACCT | cAMP-dependent protein kinase catalytic subunit |  |
|  | VmPKA2-Q-R | AGTGCCCACCAGTCTACAGC |  |  |
| VM1G_08329 | VmPKR-Q-F | CCCTCAAGCCTAATACCGA | cAMP-dependent protein kinase regulatory subunit |  |
|  | VmPKR-Q-R | AGAAATAGTCACCAGCATCACC |  |  |
