# Supplementary material for: Gα proteins Gvm2 and Gvm3 regulate vegetative growth, asexual development, and pathogenicityon apple in Valsa mali
Source: PLoS One. 2017 Mar 7;12(3):e0173141. doi: 10.1371/journal.pone.0173141 (PMC5340391; doi:10.1371/journal.pone.0173141)
Supplement: S3 Table — (DOCX) [file pone.0173141.s005.docx]

| **Table S3. Primers used for melanin biosynthesis related genes and cell wall-degrading enzyme genes expression** | | | | |
| --- | --- | --- | --- | --- |
| **Sequence number** | **Primer name** | **Sequence(5'-3')** | **Annotation** | **Function** |
| VM1G_05725 | VM05725Q-F | CTCGCCCATGTACTATGTCTTC | Exopolygalacturonase | Pectinase activity |
|  | VM05725Q-R | GTATCCCAGCCATCCGTATTC |  |  |
| VM1G_03030 | VM03030Q-F | CCCGCACTACTTCTTCTTTGA | Exopolygalacturonase C | Pectinase  activity |
|  | VM03030Q-R | ACGTCGCTTCCTTGGATTT |  |  |
| VM1G_10448 | VM10448Q-F | ACGATGGCACTACGGTTATTT | Endopolygalacturonase C | Pectinase  activity |
|  | VM10448Q-R | CCCTTCACGGTGATGTTGT |  |  |
| VM1G_04322 | VM04322Q-F | CGAGAGGACGTGGGTAAATATC | exopolygalacturonase | Pectinase  activity |
|  | VM04322Q-R | GACCGTGCGTACCATTACA |  |  |
| VM1G_06261 | VM06261Q-F | GTGACCATCTCTAACAGCCATATC | Pectate lyase | Pectinase  activity |
|  | VM06261Q-R | CTGGTCATCAGATCCCGTAAAG |  |  |
| VM1G_02197 | VM02197Q-F | TCACGATGAGCAGCATGAC | **E**ndopolygalacturonase | Pectinase  activity |
|  | VM02197Q-R | CACCAGAGGTGCAAGTAACA |  |  |
| VM1G_10963 | VM10963Q-F | CTGGACAACACTGACCTGAT | Pectate lyase | Pectinase  activity |
|  | VM10963Q-R | GAGATGGTGACACGGTTAGAG |  |  |
| VM1G_07780 | VM07780Q-F | CGGTCTGTCCATTGGCTC | endo-polygalacturonase | Pectinase  activity |
|  | VM07780Q-R | GAGATGCCAGACAGGGTGAT |  |  |
| VM1G_00119 | VM00119Q-F | TCCATTGGCTCAGTCGGTG | endo-polygalacturonase | Pectinase  activity |
|  | VM00119Q-R | GCCGCCCTTGTAGTTCTGC |  |  |
| VM1G_07103 | VM07103Q-F | CCTCAATCTCTCAACCCTCTTC | Endo-1,4-beta-xylanase | Hemicellulase activity |
|  | VM07103Q-R | CCAACACCATCAGTCCAGAA |  |  |
| VM1G_01177 | VM01177Q-F | GAGGTCATGGAGGCACATATC | Putative xylanase III | Hemicellulase activity |
|  | VM01177Q-R | GTAGAAGATGTTGTCCCGGTAAG |  |  |
| VM1G_08103 | VM08103Q-F | GGAAGAAACTGGGCATGTCTAT | Endo-1,4-beta  -xylanase | Hemicellulase activity |
|  | VM08103Q-R | CTAAGCCTCAGACACCGTAATG |  |  |
| VM1G_05031 | VM05031Q-F | CCTCCCACGAACAAGGATATT | Xylosidase : arabinofuranosidase | Hemicellulase activity |
|  | VM05031Q-R | GGGTTTGGGTATCTCCAGTATG |  |  |
| VM1G_10016 | VM10016Q-F | CCTACAAAGAGGCAACAACAAC | Galactan-beta-  galactosidase | Hemicellulase activity |
|  | VM10016Q-R | TCCGGCGGATAAACAACTATAC |  |  |
| VM1G_10306 | VM10306Q-F | GCTTCGCGGATTTACCAAAC | Beta-glucosidase cel3A | Cellulase activity |
|  | VM10306Q-R | CTGGTCTTCCACATCCCAATAA |  |  |
| VM1G_07764 | VM07764Q-F | GAGACGATCAAGGGTACACAAG | Beta-glucosidase cel3A | Cellulase activity |
|  | VM07764Q-R | CGGATTGCGTGTTGTTGTATG |  |  |
| VM1G_00613 | VM100613Q-F | CTCCCGTAGGCATTCGATTT | putative beta-glucosidase A | Cellulase activity |
|  | VM100613Q-R | CCATTGCTAGACCTCGTTGAT |  |  |
| VM1G_10301 | VM10301Q-F | CAACGAGTGGGACACTTCTATC | Putative 1,4-beta-D-glucan cellobiohydrolase B | Cellulase activity |
|  | VM10301Q-R | CTGTAGTCACTTCCATCGACAC |  |  |
| VM1G_10790 | VM10790Q-F | CATCCGAACCATCCTCATCATAG | 1,4-beta-D-glucan cellobiohydrolase CEL6A | Cellulase activity |
|  | VM10790Q-R | GGCATAGTTAACGCCCGTATAG |  |  |
| VM1G_09037 | VM09037Q-F | TGTTCAGCGTCAGTGCATATAG | Endoglucanase cel12C | Cellulase activity |
|  | VM09037Q-R | TTGGTCGTCGTGGTAGTAGT |  |  |
